# Supplementary material for: Widely Used Commercial ELISA Does Not Detect Precursor of Haptoglobin2, but Recognizes Properdin as a Potential Second Member of the Zonulin Family
Source: Front Endocrinol (Lausanne). 2018 Feb 5;9:22. doi: 10.3389/fendo.2018.00022 (PMC5807381; doi:10.3389/fendo.2018.00022)
Supplement: Supplementary file 1 [file Table_1.PDF]

	Haptoglobin genotype	Zonulin ELISA ng/ml	age years	BMI kg/m²	Kit Lot Nr.
pooled low samples	1-2	33,73	47,7	n.d.	5601 150805
	2-2	32,45	36,6	29,7	5601 150805
	1-2	32,25	39,4	20,2	5601 150805
	1-2	32,01	41,9	43	5601 150805
	1-1	30,91	46,2	23,1	5601 150805
	2-2	30,1	79,2	29,8	5601 150805
	1-2	18,02	26,5	20,9	5601 150805
	1-1	17,83	61,5	28,7	5601 150805
pooled high samples group1	2-2	207,11	47,6	28,8	5601 150623
	1-1	203,04	27,7	23,3	5601 150623
	1-2	199,2	64,4	28,2	5601 150623
	n.d.	185,45	65,5	27	5601 150623
	n.d.	173,73	55,8	32,4	5601 150623
	1-2	171,77	51,2	29,5	5601 150623
	1-2	170,26	43	28,6	5601 150623
	1-2	152,98	52,1	31,8	5601 150623
pooled high samples group2	2-2	149,55	36,8	20,1	5601 150805
	2-2	146,26	27,1	20,2	5601 150805
	1-2	143,5	44,5	36,9	5601 150805
	1-2	140,03	61,6	30,3	5601 150805
	n.d.	139,83	38,1	29,4	5601 150805
	1-2	137,65	44,8	28,5	5601 150805
	n.d.	133,49	67,7	34,9	5601 150805
	2-2	132,91	56,8	31,2	5601 150805
