# Supplementary material for: Widely Used Commercial ELISA Does Not Detect Precursor of Haptoglobin2, but Recognizes Properdin as a Potential Second Member of the Zonulin Family
Source: Front Endocrinol (Lausanne). 2018 Feb 5;9:22. doi: 10.3389/fendo.2018.00022 (PMC5807381; doi:10.3389/fendo.2018.00022)
Supplement: Supplementary file 3 [file Table_3.PDF]

Supplementary Table 3: Multiple stepwise linear regression analysis

Model		Model strength		β	T	Significance
		Adj. R^2	*Sig.*			
1	Age (years)	0.000	0.962	0.012	0.234	0.815
	Sex			0.007	0.139	0.890
2	Age	0.025	0.024	-0.058	-1.029	0.304
	Sex			0.002	0.034	0.973
	BMI			0.174	3.083	0.002
3	Age	0.092	0.000	-0.063	-1.158	0.247
	Sex			-0.078	-1.494	0.136
	BMI			0.106	1.889	0.060
	Triglycerides			0.281	5.209	0.000
4	Age	0.122	0.000	-0.106	-1.929	0.054
	Sex			-0.09	-1.751	0.081
	BMI			0.061	1.08	0.281
	Triglycerides			0.238	4.368	0.000
	Fasting glucose			0.196	3.493	0.001
5	Age	0.134	0.000	-0.139	-2.46	0.014
	Sex			-0.09	-1.768	0.078
	BMI			0.053	0.944	0.346
	Triglycerides			0.193	3.333	0.001
	Fasting glucose			0.196	3.5	0.001
	Apolipoprotein B			0.128	2.279	0.023

Adj. R^2- Adjusted coefficient of determination; Sig.- Significance (p-value) of the model; β- standardized coefficient β; T- t-value; Significance- P-value of each individual fact
