# Supplementary material for: Widely Used Commercial ELISA Does Not Detect Precursor of Haptoglobin2, but Recognizes Properdin as a Potential Second Member of the Zonulin Family
Source: Front Endocrinol (Lausanne). 2018 Feb 5;9:22. doi: 10.3389/fendo.2018.00022 (PMC5807381; doi:10.3389/fendo.2018.00022)
Supplement: Supplementary file 4 [file Table_4.PDF]

Supplementary Table 4: Publications using the analyzed ELISA kit (Pubmed search: "zonulin", last updated 07.07.2017)

Author (year)	Title	Journal
Barcelo, Antonia et al. (2016)	Gut epithelial barrier markers in patients with obstructive sleep apnea	Sleep medicine
Chwist, Alina et al. (2014)	A composite model including visfatin, tissue polypeptide-specific antigen, hyaluronic acid, and hematological variables for the diagnosis of moderate-to-severe fibrosis in nonalcoholic fatty liver disease: a preliminary study	Polskie Archiwum Medycyny Wewnetrznej.
Damms-Machado, A. et al. (2017)	Gut permeability is related to body weight, fatty liver disease, and insulin resistance in obese individuals undergoing weight reduction	The American journal of clinical nutrition
Ficek, Joanna et al. (2017)	Relationship between plasma levels of zonulin, bacterial lipopolysaccharides, D-lactate and markers of inflammation in haemodialysis patients	International urology and nephrology
Frin, Anne-Claire et al. (2016)	Accuracies of fecal calprotectin, lactoferrin, M2-pyruvate kinase, neopterin and zonulin to predict the response to infliximab in ulcerative colitis	Digestive and liver disease: official journal of the Italian Society of Gastroenterology and the Italian Association for the Study of the Liver.
Funderburg, Nicholas T. et al. (2016)	Rosuvastatin Decreases Intestinal Fatty Acid Binding Protein (I-FABP), but Does Not Alter Zonulin or Lipopolysaccharide Binding Protein (LBP) Levels, in HIV-Infected Subjects on Antiretroviral Therapy	Pathogens & immunity
Gerdes, Sasha et al. (2012)	Zonulin may not be a marker of autoimmunity in patients with psoriasis	Acta dermato-venereologica
Halasa, Maciej et al. (2017)	Oral Supplementation with Bovine Colostrum Decreases Intestinal Permeability and Stool Concentrations of Zonulin in Athletes	Nutrients
Hunt, Peter W. et al. (2014)	Gut epithelial barrier dysfunction and innate immune activation predict mortality in treated HIV infection	The Journal of infectious diseases
Iacomino, Giuseppe et al. (2016)	Protective effects of ID331 Triticum monococcum gliadin on in vitro models of the intestinal epithelium	Food chemistry
Karakula-Juchnowicz, Hanna et al. (2014)	The role of IgG hypersensitivity in the pathogenesis and therapy of depressive disorders	Nutritional neuroscience
Klaus, Daniel A. et al. (2013)	Increased plasma zonulin in patients with sepsis	Biochemia medica.
Kume, Tuncay et al. (2016)	The Relationship of Serum Zonulin Level with Clinical and Laboratory Parameters in Childhood Obesity	Journal of clinical research in pediatric endocrinology

Kuzma, Jessica N. et al. (2016)	No differential effect of beverages sweetened with fructose, high-fructose corn syrup, or glucose on systemic or adipose tissue inflammation in normal-weight to obese adults: a randomized controlled trial	The American journal of clinical nutrition
Lamprecht, Manfred et al. (2012)	Probiotic supplementation affects markers of intestinal barrier, oxidation, and inflammation in trained men; a randomized, double-blinded, placebo-controlled trial	Journal of the International Society of Sports Nutrition
Lamprecht, Manfred et al. (2015)	Effects of zeolite supplementation on parameters of intestinal barrier integrity, inflammation, redoxbiology and performance in aerobically trained subjects	Journal of the International Society of Sports Nutrition
Li, Chuanwei et al. (2016)	Zonulin Regulates Intestinal Permeability and Facilitates Enteric Bacteria Permeation in Coronary Artery Disease	Scientific reports
Lukaszyk, Ewelina et al. (2015)	Iron Status and Inflammation in Early Stages of Chronic Kidney Disease	Kidney & blood pressure research
Malyszko, Jolanta et al. (2014)	Zonulin, iron status, and anemia in kidney transplant recipients: are they related?	Transplantation proceedings
Marlicz, Wojciech et al. (2016)	Effect of colorectal cancer on the number of normal stem cells circulating in peripheral blood	Oncology reports
Mishra, Asha et al. (2016)	Structural and Functional Changes in the Tight Junctions of Asymptomatic and Serology-negative First-degree Relatives of Patients With Celiac Disease	Journal of clinical gastroenterology
Mokkala, Kati et al. (2017)	Increased intestinal permeability, measured by serum zonulin, is associated with metabolic risk markers in overweight pregnant women	Metabolism: clinical and experimental
Mokkala, Kati et al. (2017)	Evaluation of serum zonulin for use as an early predictor for gestational diabetes	Nutrition & diabetes
Mokkala, Kati et al. (2016)	Gut Microbiota Richness and Composition and Dietary Intake of Overweight Pregnant Women Are Related to Serum Zonulin Concentration, a Marker for Intestinal Permeability	The Journal of nutrition
Moreno-Navarrete, Jose Maria et al. (2012)	Circulating zonulin, a marker of intestinal permeability, is increased in association with obesity-associated insulin resistance	PloS one
Ohlsson, Bodil et al. (2017)	Higher Levels of Serum Zonulin May Rather Be Associated with Increased Risk of Obesity and Hyperlipidemia, Than with Gastrointestinal Symptoms or Disease Manifestations	International journal of molecular sciences
Ohlsson, Bodil et al. (2017)	Calprotectin in serum and zonulin in serum and feces are elevated after introduction of a diet with lower carbohydrate content and higher fiber, fat and protein contents	Biomedical reports

Ohlsson, Bodil et al. (2016)	Two meals with different carbohydrate, fat and protein contents render equivalent postprandial plasma levels of calprotectin, cortisol, triglycerides and zonulin	International journal of food sciences and nutrition
Orlando, Antonella et al. (2014)	Lactobacillus GG restoration of the gliadin induced epithelial barrier disruption: the role of cellular polyamines	BMC microbiology
Pacifico, Lucia et al. (2014)	Increased circulating zonulin in children with biopsy-proven nonalcoholic fatty liver disease	World Journal of Gastroenterology
Pärtty, Anna et al. (2016)	Infantile Colic is Associated with Low-grade Systemic Inflammation	Journal of pediatric gastroenterology and nutrition
Przybyłowski, P. et al. (2014)	Zonulin and Iron Metabolism in Heart Transplant Recipients	Transplantation proceedings
Russo, Francesco et al. (2012)	Inulin-enriched pasta improves intestinal permeability and modifies the circulating levels of zonulin and glucagon-like peptide 2 in healthy young volunteers	Nutrition research
Russo, Francesco et al. (2013)	The effects of fluorouracil, epirubicin, and cyclophosphamide (FEC60) on the intestinal barrier function and gut peptides in breast cancer patients: an observational study	BMC cancer
Serrano-Villar, Sergio et al. (2016)	Effects of Combined CCR5/Integrase Inhibitors-Based Regimen on Mucosal Immunity in HIV-Infected Patients Naive to Antiretroviral Therapy: A Pilot Randomized Trial	PLoS pathogens
Skardelly, Marco et al. (2009)	Expression of Zonulin, c-kit, and Glial Fibrillary Acidic Protein in Human Gliomas	Translational oncology
Sket, Robert et al. (2017)	Hypoxia and Inactivity Related Physiological Changes (Constipation, Inflammation) Are Not Reflected at the Level of Gut Metabolites and Butyrate Producing Microbial Community. The PlanHab Study	Frontiers in physiology
Stadlbauer, Vanessa et al. (2015)	Lactobacillus casei Shirota Supplementation Does Not Restore Gut Microbiota Composition and Gut Barrier in Metabolic Syndrome: A Randomized Pilot Study	PloS one
Stenman, Lotta K. et al. (2016)	Probiotic With or Without Fiber Controls Body Fat Mass, Associated With Serum Zonulin, in Overweight and Obese Adults-Randomized Controlled Trial	EBioMedicine
Vorobjova, Tamara et al. (2016)	Circulating Zonulin Correlates with Density of Enteroviruses and Tolerogenic Dendritic Cells in the Small Bowel Mucosa of Celiac Disease Patients	Digestive diseases and sciences.
Wex, Thomas et al. (2009)	Zonulin is not increased in the cardiac and esophageal mucosa of patients with gastroesophageal reflux disease	Peptides

Wosiewicz, Piotr et al. (2016)	Portal vein thrombosis in cirrhosis is not associated with intestinal barrier disruption or increased platelet aggregability	Clinics and research in hepatology and gastroenterology
Żak-Gołąb, Agnieszka et al. (2013)	Gut Microbiota, Microinflammation, Metabolic Profile, and Zonulin Concentration in Obese and Normal Weight Subjects	International Journal of Endocrinology
Zhang, D. et al. (2014)	Circulating zonulin levels in newly diagnosed Chinese type 2 diabetes patients	Diabetes research and clinical practice
Zhang, Dongmei et al. (2015)	Serum zonulin is elevated in women with polycystic ovary syndrome and correlates with insulin resistance and severity of anovulation	European journal of endocrinology

Note: The kit sold by other companies (e.g. ALPCO) is the same as the Immundiagnostik kit
